# Laminarin promotes anti-cancer immunity by the maturation of dendritic cells

**DOI:** 10.18632/oncotarget.16170

**Published:** 2017-03-14

**Authors:** Kyeongeun Song, Li Xu, Wei Zhang, Yun Cai, Bian Jang, Junghwan Oh, Jun-O Jin

**Affiliations:** ^1^ Shanghai Public Health Clinical Center, Shanghai Medical College, Fudan University, Shanghai, 201508, China; ^2^ Marine-Integrated Bionics Research Center, Pukyong National University, Busan, 48513, Korea; ^3^ Department of Biomedical Engineering and Center for Marine-Integrated Biomedical Technology (BK21 Plus), Pukyong National University, Busan, 48513, Korea; ^4^ Interdisciplinary Program of Biomedical Mechanical and Electrical Engineering, Pukyong National University, Busan, 48513, Korea

**Keywords:** laminarin, adjuvant, anti-cancer, dendritic cell maturation, cytotoxic lymphocyte activation

## Abstract

This research evaluates the effects of laminarin on the maturation of dendritic cells and on the *in vivo* activation of anti-cancer immunity. *In vivo* treatment of C56BL/6 mice with laminarin increased the expression levels of co-stimulatory molecules and the production of pro-inflammatory cytokines in spleen dendritic cells. Laminarin enhanced ovalbumin antigen presentation in spleen dendritic cells and promoted the proliferation of OT-I and OT-II T cells. Laminarin also induced the maturation of dendritic cells in tumor-draining lymph nodes and protected interferon-γ and tumor necrosis factor-α and proliferation of OT-I and OT-II T cells in tumors. The combination treatment of laminarin and ovalbumin inhibited B16-ovallbumin melanoma tumor growth and its liver metastasis by antigen-specific immune activation, including cytotoxic T lymphocyte activation and interferon-γ production. Thus, these data demonstrated the potential of laminarin as a new and useful immune stimulatory molecule for use in cancer immunotherapy.

## INTRODUCTION

Dendritic cells (DCs) are antigen (Ag) presenting cells (APCs) that, after exposure to stimuli, undergo maturation, which presents as increased levels of co-stimulatory molecules and pro-inflammatory cytokine production and Ag presentation in T cells [1]. Matured DCs activate adaptive immune responses, including T helper (Th) and cytotoxic T lymphocyte (CTL) activation [1, 2]. In mice, there are two primary populations of DCs: plasmacytoid DCs (pDCs) and conventional DCs (cDCs). Of these, cDCs are further divided two main populations, CD8α^+^CD11c^+^ and CD8α^−^CD11c^+^, which display different abilities for Ag presentation and T cell activation [3]. CD8α^+^CD11c^+^ cDCs have the selective ability to cross-present exogenous Ags in major histocompatibility complex (MHC) class I molecules and induce activation of CD8 T cells [4]. In contrast, CD8α^−^CD11c^+^ cDCs that make up the majority of murine cDCs capture extracellular Ags and present these Ags to CD4 T cells by creating a compound with the MHC class II [5].

Effective cancer immunotherapy induces cancer Ag-specific immune responses that kill cancer cells [6, 7]. Ag-specific CTL activation and antibody production against cancer Ags are the most common strategies to effectively kill cancer cells [6, 7]; however, in the tumor microenvironment, the interaction between cancer cells and immune cells leads to immune suppression, which exhibits as tumor growth and preserves tumor cells from immune responses [8]. In addition, cancer Ags are not enough to induce Ag-specific CTL activation and antibody production because DCs and macrophages present low levels of cancer Ags and express low levels of co-stimulatory molecules [9, 10]. Therefore, adjuvant components, which strongly promote maturation of DCs, are required together with cancer Ags to enhance tumor immunity [6, 11].

Polysaccharides, including laminarin, fucoidan, and alginate from brown seaweed, present effective functions of biological activities [12–14] and promote activation of immune cells, such as DCs, macrophages, natural killer (NK) cells, T cells and B cells, and enhance anti-viral and anti-tumor responses [15, 16]. Laminarin is a polysaccharide extracted from brown seaweed and composed of (1→3)-beta-D-glucan and (1→6)-beta-linkage [17, 18]. Previous studies have demonstrated that laminarin induces activation of RAW 264.7 cells, the murine macrophage cell line, and bone-marrow-derived DCs (BMDCs) [19, 20], and the activation of BMDCs by laminarin-promoted Ag-specific CD4 T cell activation was achieved *in vitro* [20]. Although the effects of laminarin on DC activation has been investigated *in vitro*, *in vivo* DC activation and the adjuvant effects of laminarin to induce anti-cancer immunity through Ag-specific immune activation have not been investigated. In this study, *in vivo* administration of laminarin to induce DC maturation and Ag-specific T cells to initiate anti-cancer effects and the effectiveness of laminarin functions as an adjuvant for the treatment of B16 melanoma in mouse model was investigated *in vivo*.

## RESULTS

### Laminarin-induced maturation of spleen DCs

**Figure 1:**
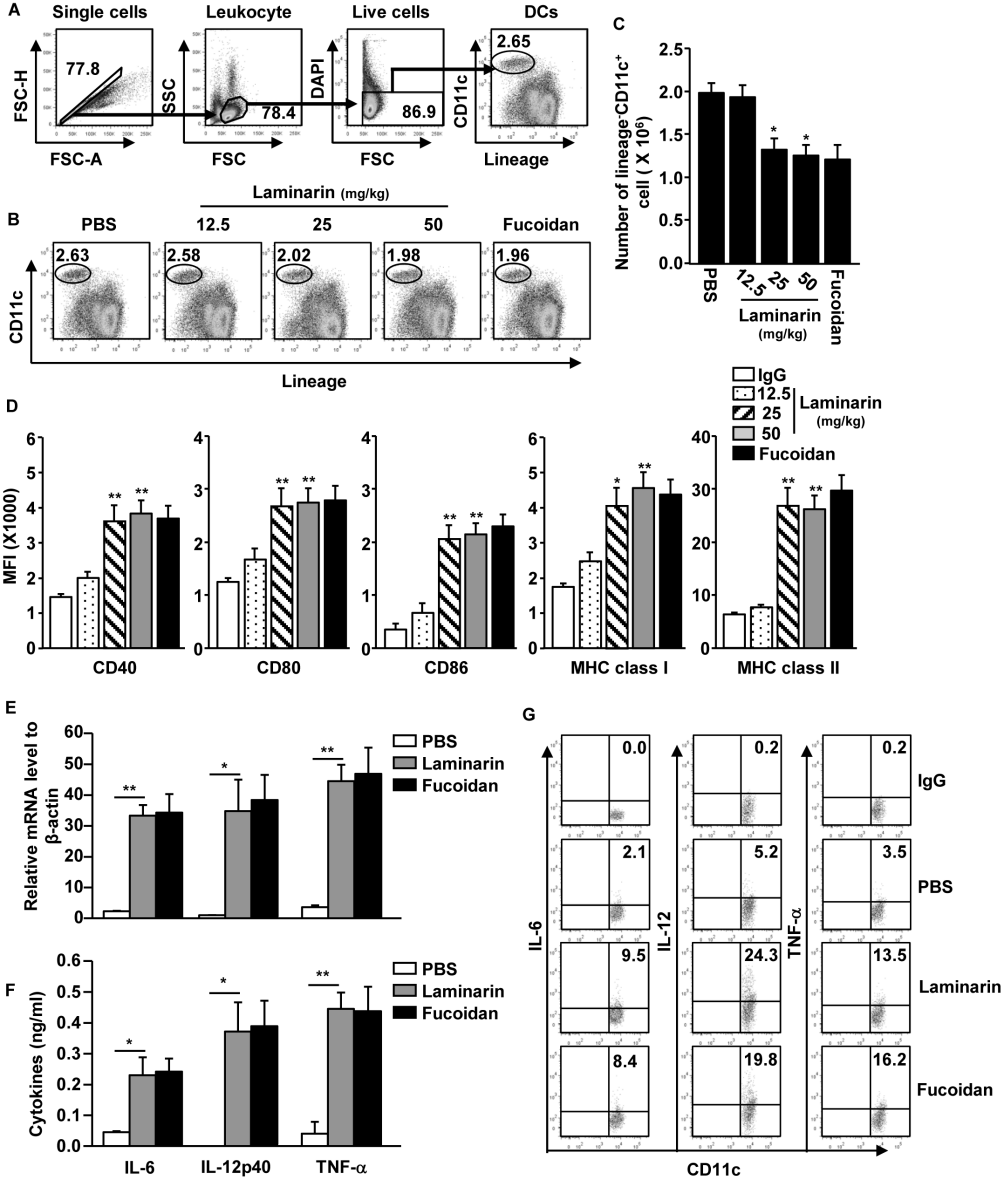
Laminarin-induced activation of spleen DCs *in vivo* C57BL/6 mice were injected intravenously (*i.v*.) with 12.5, 25, and 50 mg/kg of laminarin and 10 mg/kg of fucoidan for 24 hours before the spleens were harvested. **(A)** Definition of spleen DCs. Lineage markers included CD3, Thy1.1, B220, Gr-1, CD49b, and TER-119. **(B)** Percentages of lineage^−^CD11c^+^ DCs in the spleens. **(C)** Mean of the absolute numbers of lineage^−^CD11c^+^ spleen DCs within live cells. **(D)** Mean fluorescence intensity (MFI) of co-stimulatory molecules and MHC classes I and II in gated lineage^−^CD11c^+^ cells from the spleens were analyzed using flow cytometry. **(E)** mRNA levels. **(F)** Sera concentration of IL-6, IL-12p70, and TNF-α. **(G)** Intracellular IL-6, IL-12, and TNF-α production in spleen DCs. All data are representative of or the average of analyses of six independent samples (two mice per experiment, totaling three independent experiments). *p < 0.05, **p < 0.01.

Because laminarin promotes macrophage and DC activation *in vitro* [19, 20], the ability of laminarin to induce spleen DC activation *in vivo* was studied. Thus, C57BL/6 mice were injected intravenously (*i.v*.) with 12.5, 25, and 50 mg/kg of laminarin, and 10 mg/kg of fucoidan as a positive control. Twenty-four hours after injection, the activation of spleen DCs was analyzed using flow cytometry. The spleen DCs were defined as lingeage^−^CD11c^+^ cells in DAPI^−^live cells (Figure 1A), and treatments of 25 and 50 mg/kg of laminarin decreased the frequency and number of spleen DCs (Figure 1B and 1C), while the expression levels of co-stimulatory molecules and MHC class I and II were significantly increased (Figure 1D). The elevation of co-stimulatory molecules and MHC class I and II levels were similar to those achieved by fucoidan induction, which is a marine-derived polysaccharide with well-defined effects promoting DC maturation (Figure 1B, 1C, and 1D).

Activated DCs produce pro-inflammatory cytokines; thus, whether laminarin can promote production of pro-inflammatory cytokines in spleen DCs was investigated. C57BL/6 mice were injected *i.v*. with 25 mg/kg of laminarin for 2, 4, or 24 hours, and mRNA expression levels of interlukin-6 (IL-6), IL-12p40, and tumor necrosis factor-α (TNF-α) were dramatically increased 2 hours after laminarin injection (Figure 1E). In addition, serum concentrations of IL-6, IL-12p40, and TNF-α substantially increased 24 hours after treatment with laminarin (Figure 1F). To determine whether laminarin-activated spleen DCs contributed to increases in pro-inflammatory cytokine production, intracellular cytokine production in spleen DCs was examined by injecting mice with 25 mg/kg of laminarin for 4 hours and incubating splenocytes in a monensin solution for 4 hours. Intracellular IL-6, IL-12p40, and TNF-α levels were substantially up-regulated after treatment with laminarin (Figure 1G), and consistent with co-stimulatory expression levels, laminarin-induced production levels of pro-inflammatory cytokines were similar to those induced with fucoidan. Thus, these data suggest that laminarin-induced activation of spleen DCs *in vivo* up-regulate co-stimulatory molecule expression and produce pro-inflammatory cytokines.

### Laminarin-induced Th1 and Tc1 responses *in vivo*

**Figure 2:**
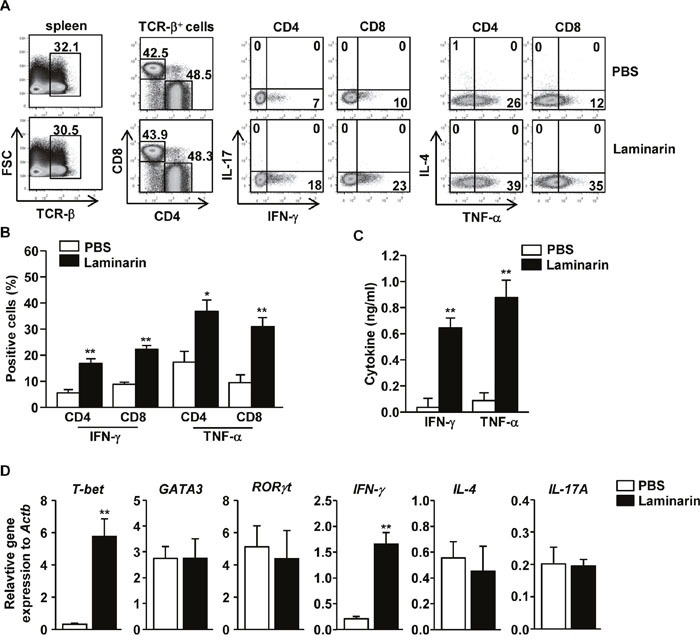
Laminarin-promoted Th1 and Tc1 responses *in vivo* C57BL/6 mice were injected *i.v*. with 25 mg/kg of laminarin for 3 days. The mice were injected again with the same amount of laminarin for an additional 3 days. **(A)** Percentage of IFN-γ, IL-17, IL-4, and TNF-α-producing cells within CD4 and CD8 T cells in the spleens, assessed using a flow cytometric analysis. **(B)** Mean percentages of IFN-γ- or TNF-α-producing CD4 and CD8 T cells. **(C)** Concentrations of IFN-γ and TNF-α in the mice sera. All data are representative of or the average of analyses of six independent samples (two mice per experiment, for a total of three independent experiments). **(D)** Gene expression in the spleens was measured 24 hours after laminarin injection. Data are the average of analyses of six independent samples (two mice per experiment, for a total of three independent experiments). *p < 0.05, **p < 0.01.

Because laminarin induces activation of spleen DCs, laminarin-activated spleen DCs that subsequently promote CD4 and CD8 T cell responses *in vivo* were also studied. Mice were injected *i.v*. with 25 mg/kg of laminarin, which was repeated after three days. Treatment with laminarin promoted marked increases in the proportion of interferon-γ- (IFN-γ)- and TNF-α-producing CD4 and CD8 T cells, whereas T cells did not produce IL-4 and IL-17 (Figure 2A and 2B). Moreover, serum concentrations of IFN-γ and TNF-α also increased significantly after laminarin treatment (Figure 2C). Laminarin-treated splenocyte expressed significantly higher mRNA levels of T-bet (*p < 0.01*), which is a critical transcription factor for Th1 and Tc1 cells, and IFN-γ (*p < 0.01*) compared to the phosphate-buffered saline (PBS)-treated control (Figure 2D). In contrast, the mRNA levels of IL-4, IL-17A, GATA3, and RORγt, which are transcription factors for Th2 and Th17, were not altered by treatment with laminarin (Figure 2D). Therefore, these data suggest that laminarin-activated DCs promote Th1 and Tc1 responses *in vivo*.

### Laminarin-enhanced, Ag-specific immune responses

**Figure 3:**
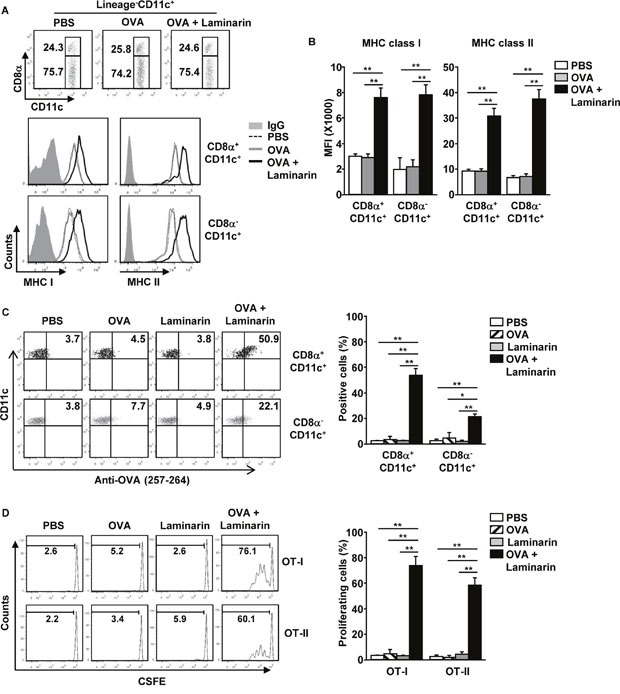
Laminarin-induced, Ag-specific immune responses C57BL/6 mice were injected *i.v*. with PBS, 50 μg of OVA, 25 mg/kg of laminarin, and the combination of laminarin and OVA for 24 hours. **(A)** CD8α^+^ and CD8α^−^ cDCs in the spleens and CD11c^+^ DCs (upper panel). Expression levels of MHC classes I and II were analyzed using flow cytometry (low panel). **(B)** MFI of MHC classes I and II in the CD8α^+^ and CD8α^−^ cDCs. **(C)** Surface OVA peptide (257-264) presentation was measured in CD8α^+^ and CD8α^−^ cDCs (left panel). Mean percentage of OVA peptide (257-264) positive cells in the CD8α^+^ and CD8α^−^ cDCs (right panel). **(D)** Proliferation of adaptive transferred CFSE-labeled OT-I and OT-II T cells in the CD45.1 congenic mice were analyzed using flow cytometry (left panel). Mean percentage of proliferating cells (right panel). Data are the average of analyses of six independent samples (two mice per experiment, for a total of three independent experiments). *p < 0.05, **p < 0.01.

To determine whether laminarin induces Ag-specific immune responses, C57BL/6 mice were injected *i.v*. with a combination of 25 mg/kg of laminarin and 50 μg of ovalbumin (OVA), and MHC class I and II expression levels in spleen CD8α^+^ and CD8α^−^ cDCs were examined. It was found that these cDCs were substantially up-regulated by the combination of laminarin and OVA 24 hours after treatment (Figure 3A and 3B). The capacity for Ag presentation in laminarin-stimulated spleen CD8α^+^ and CD8α^−^ cDCs was also examined. Since there is only one commercially available anti-OVA peptide (257-264) antibody for detecting OVA peptide presentation, OVA peptide (257-264) presentation in spleen CD8α^+^ and CD8α^−^ cDCs was measured. As shown in Figure 3C, the combination of laminarin and OVA treatment led to significant increases in the percentage of OVA peptide (257-264) positive CD8α^+^ and CD8α^−^ cDCs, which indicated that these cDCs presented OVA peptide (257-264) on the surface. In contrast, OVA or laminarin treatments did not induce Ag presentation in these DCs. To further determine laminarin promotion of Ag-specific immune responses, 5(6)-Carboxyfluorescein N-hydroxysuccinimidyl ester (CFSE)-labeled OT-I and OT-II cells were transferred into CD45.1 congenic mice, and 24 hours later, the mice received PBS, OVA, laminarin, and a combination of laminarin and OVA for 3 days. The proliferation of OT-I and OT-II was substantially up-regulated by the combined laminarin and OVA treatment, whereas OVA or laminarin alone did not increase proliferation of OT-I and OT-II cells (Figure 3D). Thus, these data suggest that laminarin-enhanced Ag presentation in DCs promote Ag-specific T cell responses *in vivo*.

### Laminarin-induced maturation of DCs in a tumor microenvironment

**Figure 4:**
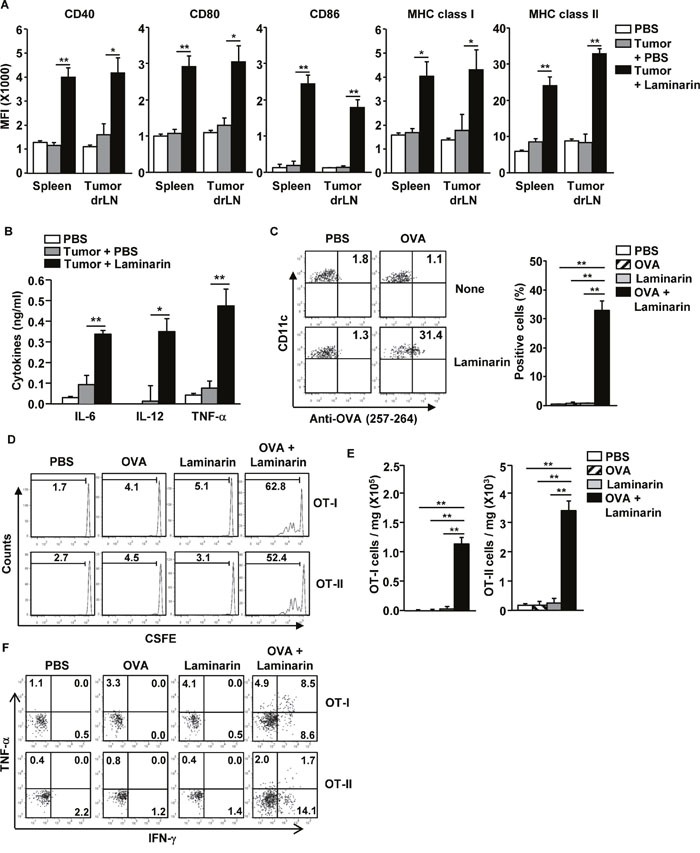
Laminarin-promoted maturation of drLNs and DCs in the tumor microenvironment C57BL/6 mice were inoculated *s.c*. with 1 × 10^6^ B16 melanoma cells or B16-OVA cells. Fifteen days after tumor injection, the mice were treated with PBS and 25 mg/kg of laminarin for 24 hours. **(A)** MFI of CD40, CD80, CD86, and MHC classes I and II levels were measured in the spleen and tumor drLNs and DCs. **(B)** Concentrations of IL-6, IL-12p40, and TNF-α in the mice sera. **(C)** Surface OVA peptide (257-264) presentation was measured in the tumor drLNs and DCs. **(D)** CFSE-labeled OT-I and OT-II T cell proliferation in B16-OVA tumor-bearing CD45.1 congenic mice were analyzed using flow cytometry. **(E)** The means of the absolute numbers of OT-I (left panel) and OT-II (right panel) cells in the tumor. **(F)** Percentage of IFN-γ^+^ and TNF-α^+^ cells in tumor-infiltrated OT-I and OT-II cells. All data are representative of or the average of analyses of six independent samples (two mice per experiment, for a total of three independent experiments). **p < 0.01, *p < 0.05.

To evaluate the maturation effects of laminarin on DCs in a tumor microenvironment, C57BL/6 mice were injected subcutaneously (*s.c*.) with 1 × 10^6^ B16 melanoma cells. Fifteen days after tumor cell injection, the mice were treated with 25 mg/kg of laminarin for 24 hours, and maturation of DCs in tumor draining lymph nodes (drLN) and spleens were examined. Treatment with laminarin induced significant increases in the CD40, 80, and 86 and MHC classes I and II expression levels in tumor drLNs and spleen DCs (Figure 4A). In addition, treatment with laminarin among tumor-bearing mice increased production of IL-6, IL-12p40, and TNF-α in serum (Figure 4B).

Next, laminarin induction of Ag-specific immune responses in the tumor microenvironment was examined. The combined laminarin and OVA treatment enhanced presentation of the OVA peptide in tumor drLNs and DCs compared to PBS, OVA, and laminarin separately treated controls (Figure 4C). The combination treatment strongly promoted OT-I and OT-II cell proliferation in B16-OVA tumor-bearing CD45.1 congenic mice (Figure 4D), and tumor infiltration of OT-I and OT-II cells in B16-OVA tumors was significantly increased by the combined treatment compared to PBS, OVA, and laminarin treatments (Figure 4E). In addition, the infiltrated OT-I and OT-II cells in the B16-OVA tumor produced remarkably higher levels of IFN-γ and TNF-α compared to the singular treatments (Figure 4F). These data suggest that laminarin-induced maturation of DCs in tumor environments including increased levels of co-stimulatory molecule expression, pro-inflammatory cytokine production, Ag presentation, and Ag-specific T cell activation.

### The laminarin and OVA combination treatment prevented B16-OVA tumor growth by inducing Ag-specific immune responses

**Figure 5:**
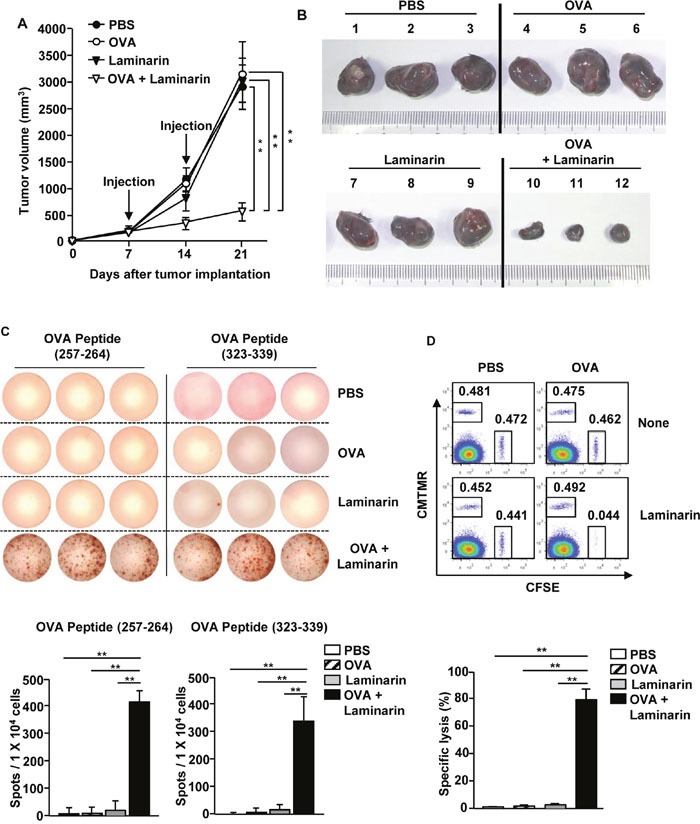
The combined laminarin and OVA treatment inhibited B16-OVA tumor growth C57BL/6 mice were injected *s.c*. with 1 × 10^6^ B16-OVA cells on the right side. Once tumors were well established on day 7, mice were injected *i.v*. with PBS, 50 μg of OVA, 25 mg/kg of laminarin, and the combination of laminarin and OVA, and 7 days later, the mice were treated with the same amount of OVA and laminarin again. **(A)** B16-OVA tumor growth curves on the right side of the mice. **(B)** The sizes of the tumor masses on day 21 after the B16-OVA tumor cell challenge. **(C)** OVA peptides (257-264) and (323-339) specific IFN-γ production in splenocytes was analyzed by ELISPOT analysis (upper panel), and the mean number of spots (lower panel). **(D)** CTL activity was assessed *in vivo* on day 21 after tumor injection by adoptively transferring splenocyte populations labeled with CFSE and loaded with SIINFEK with a control splenocyte population without a peptide labeled with CMTMR. Dot plots show the percentage of SIINFEK-loaded CFSE^+^ cells and non-peptide-loaded CMTMR^+^ cells (upper panel) and mean percentages of Ag specific lysis (lower panel). Data are from analyses of six individual mice (three mice per experiment, for a total of two independent experiments). **p < 0.01.

Laminarin-promoted maturation of DCs and activation of T cells in tumor microenvironments prompted examination of related anti-cancer effects. C57BL/6 mice were injected *s.c*. with 1 × 10^6^ B16-OVA cells. Once tumors were well established on day 7, the mice were treated *i.v*. with PBS, OVA (50 μg), laminarin (25 mg/kg), and a combination of laminarin (25 mg/kg) and OVA (50 μg). Seven days after treatment, the mice received with the same amount of OVA and laminarin, and tumor sizes were monitored during treatment. The combination treatment substantially prevented B16-OVA tumor growth, whereas OVA and laminarin treatments did not inhibit tumor growth (Figure 5A). On the 21^st^ day after treatment, the sizes of the tumor masses among mice treated with the combination treatment were significantly smaller than the tumor masses among the PBS-, OVA-, and laminarin-treated mice (Figure 5B). In addition, OVA specific IFN-γ production in splenocytes was examined using an ELISPOT analysis, and it was found that the combination of laminarin and OVA significantly increased the number of plots compared to PBS-, OVA-, and laminarin-treated splenocytes, which indicated that splenocytes from the mice given the combination treatment produced large amounts of IFN-γ in response to OVA peptides (257-264) and (323-339) compared to the mice treated with only PBS, OVA, or laminarin (Figure 5C). Furthermore, an *in vivo* cytotoxicity assay showed that specific lysis of OVA-pulsed target cells was approximately 80% in mice given the combination treatment. In contrast, OVA-pulsed target cells were not eliminated to a significant amount in the mice treated only with PBS, OVA, or laminarin (Figure 5D). These data suggest that the combination treatment induced *in vivo* anti-cancer effects in mice by activating Ag specific immune responses.

### Combined laminarin and OVA treatments inhibit liver metastasis of B16-OVA melanoma cells

**Figure 6:**
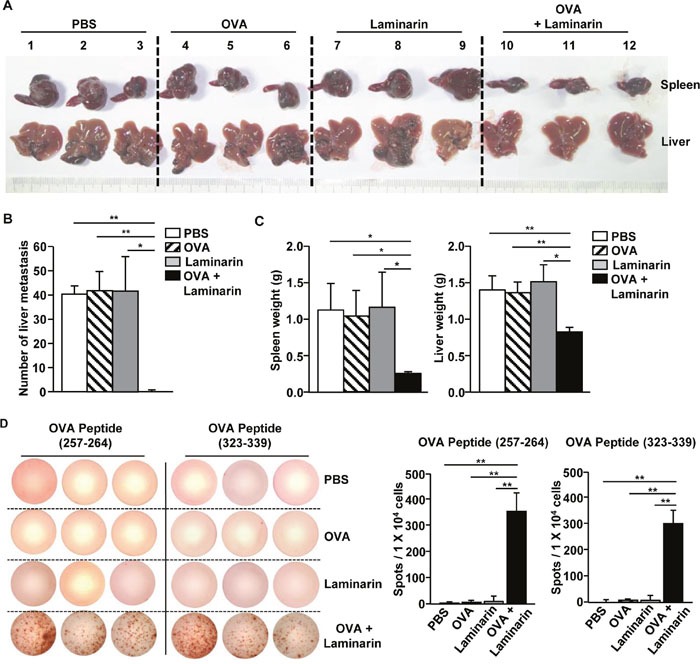
Treatment with laminarin and OVA inhibited liver metastasis of B16-OVA tumor cells C57BL/6 mice were treated *i.v*. with PBS, 50 μg of OVA, 25 mg/kg of laminarin, and the combination of laminarin and OVA. Three days after the treatment, the mice were inoculated *i.s*. with B16-OVA melanoma cells. On day 3 after the B16-OVA cell challenge, the mice again received the same amount of the laminarin and OVA treatment. **(A)** The size of the tumor masses in the spleens and liver metastasis of B16-OVA cells on day 14 after tumor injection. **(B)** The mean of the absolute number of B16-OVA metastasis in the livers. **(C)** Mean weights of spleens (left panel) and livers (right). **(D)** Splenocytes were harvested without tumor cells. OVA peptide-specific IFN-γ production in the splenocytes was analyzed using ELISPOT analysis (left panel). The mean number of IFN-γ positive spots (right panel). All data are representative of or the average of analyses of six independent samples (three mice per experiment, for a total of two independent experiments). *p<0.01, **p < 0.01.

The effects of the combination of laminarin and OVA on tumor metastasis in mice were also investigated. C57BL/6 mice were treated *i.v*. with PBS, OVA (50 μg), laminarin (25 mg/kg), and a combination of laminarin (25 mg/kg) and OVA (50 μg). Three days after treatment, the mice were inoculated intrasplenically (*i.s*.) with 0.5 × 10^6^ B16-OVA melanoma cells, and 3 days after this, the mice were treated *i.v*. with the same amount of OVA and laminarin. Fourteen days after tumor injection, the mice were killed and the spleens and livers were harvested. The combination treatment dramatically inhibited tumor growth in the spleen and tumor metastasis in the liver compared to PBS, OVA, or laminarin (Figure 6A and 6B). Consistent with tumor sizes in the spleen and metastasis in the liver, the weight of the spleens and livers were significantly lower among the mice treated with a combination of laminarin and OVA compared to mice only treated with PBS, OVA, and laminarin (Figure 6C). The splenocytes isolated from the mice given the combination treatment significantly increased the amount of IFN-γ in response to OVA peptides (257-264) and (323-339), whereas PBS, OVA, and laminarin alone did not induce IFN-γ production in splenocytes (Figure 6D). These data suggest that the combination of laminarin and OVA inhibited metastasis of melanoma cells by activating Ag specific immune responses.

## DISCUSSION

Because cancer microenvironments promote immune suppression against cancer Ags, cancer immunotherapy remains a challenging task [7, 11, 21]. In the cancer microenvironment, cancer cells induce immune suppression, which interrupts the cancer Ag presentation capacity of DCs, consequently reducing cancer Ag specific Th and CTL activation [9, 22]. Therefore, an effective adjuvant that can induce maturation of DCs and boost cancer Ag specific immune responses in the cancer microenvironment is required for efficient cancer immunotherapy [6, 21, 23]. The findings of the present study indicate that laminarin-induced maturation of DCs in the spleens and tumor drLNs of the mice promoted OVA Ag specific Th and CTL activation. Furthermore, the combination of laminarin and OVA effectively prevented B16-OVA tumor cell growth *in vivo* by activating OVA specific immune responses. These data suggest that laminarin might be an effective adjuvant for cancer immunotherapy.

Natural polysaccharides extracted from marine products, including fucoidan, ascophyllan, and λ-carrageenan, have been shown to activate immune responses in *in vivo* mice models, especially maturation of DCs and activation of CTLs [14, 24–26]. In the present study, laminarin, a polysaccharide purified from *Laminaria digitata*, was found to induce maturation of DCs *in vivo*. Laminarin is also well known as an antagonist of Dentin-1 [27]. Previous studies have shown that laminarin blocks Dectin-1 binding of particulate β (1-3)-glucans without stimulating downstream signaling [27–29]. However, other studies have shown that laminarin directly promotes immune cell activation by stimulating Dectin-1 [19, 20, 30, 31]. Fucoidan, a marine-derived polysaccharide, is a well-known antagonist of scavenger receptor-A (SR-A), which blocks signaling pathways [32–34]; however recent studies have found that fucoidan can induce DC and macrophage activation [14, 35–37]. Therefore, the antagonistic effect of laminarin still requires further investigation because the antagonistic effects of laminarin have not been examined for DCs [27–29], which express much higher levels of Dectin-1 on the surface compared to macrophages [38]. In addition, activation and binding of Dectin-1 with toll-like receptor-2 (TLR-2) causes intracellular signaling transduction [38]. Therefore, the antagonistic effects of laminarin in relation to the TLR2/6 signaling pathway in DCs must be investigated. Future research will investigate the effects of laminarin on Dectin-1 knockout, TLR-2 knockout, and Dectin-1/TLR-2 double knockout mice to determine whether laminarin can induce spleen DC activation.

Our data showed that laminarin treatment induced decreases in the spleen DC number. In contrast with that the DC number in tumor drLN and spleen in tumor-bearing mice were significantly increased (Supplementary Figure 1). Since DCs cannot proliferate, this increase of DC numbers may be a result of augmented DC migration into the spleen and tumor drLNs [39, 40]. In tumor microenvironment, inhibited chemokine expression impairs DC migration to the spleen [39, 40]. Therefore, the different alteration of DC numbers by laminarin in the naïve and tumor-bearing mice may due to the tumor microenvironment promoted different chemokine levels in the mice. We speculate that laminarin may induce elevation of chemokine expression and promote DC migration to spleen and tumor drLNs in tumor-bearing conditions. In our further studies, we will examine the effect of laminarin on the expression of chemokines and their receptors in lymphoid tissues and DCs in the tumor-bearing mice.

Cancer cell interaction with immune cells promotes immune tolerance against cancer Ags [8], and cancer Ags are not fully presented by APCs in tumor microenvironments, which consequently inhibits activation of adaptive immune cells and promotes tumor growth [8, 9, 22]. Adjuvants for cancer immunotherapy induce cell-mediated immune response in response to cancer Ags to effectively kill cancer cells in tumor microenvironments [6, 21, 41]. In this study, laminarin-induced maturation of spleen and tumor drLNs and DCs in *in vivo* tumor microenvironments were investigated, and laminarin-induced maturation of both CD8α^+^ and CD8α^−^ cDCs promoted Ag specific Th1 and CTL immune responses. Because the CTL immune response has been sought to effectively kill cancer cells, CD8α^+^ cDC activation and maturation has been demonstrated as a promising strategy for cancer immunotherapy [6, 7, 41]. In line with this, laminarin-promoted CD8α^+^ cDCs and Ag specific CTL activation might induce specific killing of OVA-pulsed splenocytes and OVA-expressing B16 melanoma cells *in vivo*.

Circulating cancer cells that induce recurrence and metastasis of cancer are the most difficult to treat and a major cause of mortality among cancer patients [42]. Because Ag specific CTL immune responses can find and eliminate Ag-expressing cancer cells [6], Ag specific CTL activation is a powerful mechanism for killing circulating cancer cells. The present study found that the combination of laminarin and OVA inhibited metastasis of B16-OVA melanoma cells in mice livers *in vivo* by activating OVA specific IFN-γ production. Moreover, the combination of laminarin and OVA induced specific killing of OVA-pulsed splenocytes in tumor-bearing mice, which indicates that this combination promoted OVA specific CTL activation. These data suggest that laminarin-induced, OVA-specific CTL and Th1 immune responses may be able to kill circulating cancer cells and prevent cancer metastasis *in vivo*.

Previous studies have shown that polysaccharide promotes immunomodulatory effects. Interestingly, oral administration of polysaccharide induces anti-inflammatory immune responses [43, 44], while systemic injection such as *i.v*. or *i.p*. of polysaccharide promotes pro-inflammatory immune responses [24–26, 45]. It also has been shown that oral administration of lipopolysaccharide (LPS) cannot induce systemic immune responses, which LPS is the most powerful immune stimulatory molecules isolated from gram negative bacteria [46, 47]. Therefore, dependent on injection route, immune modulation effect of polysaccharide may be changed. We will examine the oral administration of laminarin effect in next study whether the laminarin can induce immune activation or suppression in the mouse.

In conclusion, these results provide several lines of evidence that laminarin is a novel immune-stimulating reagent that can induce DC maturation and Ag specific Th1 and CTL activation that can effectively kill Ag-expressing B16 melanoma cells *in vivo*. The immune stimulatory function of laminarin will be potentially useful for developing tumor immunotherapy reagents for human use.

## MATERIALS AND METHODS

### Mice and cell lines

Six-week-old C57BL/6 mice, OT-I and OT-II TCR transgenic mice, and C57BL/6-Ly5.1 (CD45.1) congenic mice were obtained from the Shanghai Public Health Clinical Center and kept under pathogen-free conditions. The mice were maintained at a controlled temperature of 20–22°C, humidity of 50–60%, and lighting of 12 h: 12 h, with free access to standard rodent chow and water. All experiments were carried out in agreement with the guidelines of the Institutional Animal Care and Use Committee at the Shanghai Public Health Clinical Center. The protocol was approved by the Committee on the Ethics of Animal Experiments for the Shanghai Public Health Clinical Center (Mouse Protocol Number: SYXK-2010-0098). Mice were killed by CO_2_ inhalation euthanasia, and all efforts were made to minimize suffering. The murine melanoma cell line, B16F10 (ATCC, CRL-6475), expressing OVA (B16-OVA) was cultured in RPMI 1640 (Sigma Aldrich, 10% fetal bovine serum [FBS], 2 mM glutamine, 1 M HEPES, 100 μg/ml streptomycin, 100 U/ml penicillin, and 2 mM 2-mercaptoethanol). All cell lines were cultured at 37°C in a humidified atmosphere of 5% CO_2_ and air.

### Chemicals and cytokines

Laminarin derived from *Laminaria digitata* was purchased from Invivogen, and chicken OVA was obtained from Sigma-Aldrich. Laminarin and OVA solutions were passed through an endotoxin-removal column (Detoxi-gel: Thermo Fisher Scientific) and subsequently filtered through an endotoxin removal filter (Zetapor Dispo: Wako). The endotoxin levels in the purified laminarin were evaluated using a Limulus amebocyte lysate (LAL) assay kit (Lonza). OVA peptide 257–264 (SIINFEKL) and OVA 323-339 (ISQAVHAAHAEINEAGR) were purchased from China Peptides (China).

### Antibodies

Isotype control antibodies (Abs; IgG1, IgG2a, or IgG2b), CD11c (HL3), CD4 (GK1.5), CD8α (YTS169.4), CD40 (3/23), CD80 (16-10A1), CD86 (GL-1), anti-IL-4 (11B11), anti-IL-6 (MP5-20F3), and anti-IL-12/23p40 (C17.8) were obtained from BioLegend. Anti-MHC class I (AF6-88.5.3), anti-MHC class II (M5/114.15.2), anti-IFN-γ (XMG1.2), anti-IL-17 (TCC11-18H10.1), and anti-TNF-α (MP6-XT22) were obtained from eBioscience.

### Flow cytometry analysis

Cells were washed with PBS containing 0.5% BSA, pre-incubated for 15 min with unlabeled isotype control Abs, and labeled with fluorescence-conjugated Abs by incubation on ice for 30 min followed by washing with PBS. Cells were analyzed using a FACS Aria II (Becton Dickinson) and FlowJo 8.6 software (Tree Star). Cellular debris was excluded from the analysis by forward- and side-scatter gating. Dead cells were excluded by 4′,6-diamidino-2-phenylindole (DAPI; Sigma-Aldrich) staining and gating the DAPI-negative population. As a control for nonspecific staining, isotype-matched irrelevant monoclonal Abs (mAbs) were used.

### DC analysis

Spleen and tumor drLNs and DCs were analyzed as described by previous research [26, 48]. The tissues were cut into small fragments and digested, with 2% FBS containing collagenase for 20 min at room temperature. Cells from the digest were centrifuged into a pellet, and the pellet was re-suspended in 5 mL of a 1.077 histopaque (Sigma-Aldrich). Additional histopaque was layered below and EDTA-FBS was layered above the cell suspension, which was then centrifuged at 1700 g for 10 min. The light density fraction (< 1.077 g/cm^3^) was collected and incubated for 30 min with the following FITC-conjugated mAbs: anti-CD3 (17A2), anti-Thy1.1 (OX-7), anti-B220 (RA3-6B2), anti-Gr1 (RB68C5), anti-CD49b (DX5), and anti-TER-119 (TER-119). The lineage^−^CD11c^+^ cells were defined as cDCs, which were further divided into CD8α^+^ and CD8α^−^ cDCs. Analysis was carried out on a FACS Aria II (Becton Dickinson).

### *Ex vivo* T cell stimulation and intracellular cytokine staining

As described in detail previously [49], a single cell suspension prepared from spleen and tumor cells were stimulated *in vitro* for 4 hours with phorbol 12-myristate 13-acetate (50 ng/ml) and ionomycin (1 μM; both from Calbiochem), with the addition of monensin solution (Biolegend) during the final 2 hours. For intracellular cytokine staining, surface molecules were stained first, then fixed and permeabilized with a Cytofix/Cytoperm buffer (eBioscience) and subsequently incubated with anti-cytokine Abs in a Perm/Wash buffer (eBioscience) for 30 min. Control staining with isotype control immunoglobulin Gs (IgGs) was performed during all experiments.

### Enzyme-linked immunosorbent assay (ELISA)

IL-6, IL-12p70, IFN-γ, and TNF-α concentrations in sera were measured in triplicate using standard ELISA kits (Biolegend).

### Real-time polymerase chain reaction (PCR)

Total RNA was reverse-transcribed into cDNA using Oligo (dT) and M-MLV reverse transcriptase (Promega). The cDNA was subjected to real-time PCR amplification (Qiagen) for 40 cycles with annealing and an extension temperature of 60°C, on a LightCycler 480 real-time PCR system (Roche). Primer sequences were as follows: mouse β-actin forward, 5′-TGGATGACGATATCGCTGCG-3′; reverse, 5′-AG GGTCAGGATACCTCTCTT-3′; IL-6 forward, 5′-AAC GATGATGCACTTGCAGA-3′; reverse, 5′-GAGCA TTGGAAATTGGGGTA-3′; IL-12p40 forward, 5′-CAC ATCTGCTGCTCCACAAG-3′; reverse, 5′-CCGTCCG GAGTAATTTGGTG-3′; TNF-α forward, 5′-CCTTTC ACTCACTGGCCCAA-3′; reverse, 5′-AGTGCCTCT TCTGCCAGTTC-3′; T-bet forward, 5′-CAACAACC CCTTTGCCAAAG-3′; reverse, 5′-TCCCCCAAGCATT GACAGT-3′; GATA3 forward, 5′-CGGGTTCGGAT GTAAGTCGAGG-3′; reverse, 5′-GATGTCCCTGCTC TCCTTGCTG-3′; RORγt forward, 5′-CCGCTGAGAGG GCTTCAC-3′; reverse 5′-TGCAGGAGTAGGCCACA TTACA-3′; IFN-γ forward, 5′-GGATGCATTCATGAG TATTGC-3′; reverse, 5′-CTTTTCCGCTTCCTGAGG-3′; IL-4 forward, 5′-ACAGGAGAAGGGACGCCAT-3′; reverse 5′-GAAGCCCTACAGACGAGCTCA-3′; IL-17A forward, 5′-GCGCAAAAGTGAGCTCCAGA-3′; and reverse 5′-ACAGAGGGATATCTATCAGGG-3′.

### Tumor treatment

C57BL/6 mice were treated *i.v*. with phosphate-buffered saline (PBS), 50 μg of OVA in PBS, and 25 mg/kg of laminarin or OVA mixed with laminarin in PBS on days 7 and 14 after a B16-OVA challenge. On day 21, the mice were killed, and splenocytes were harvested for further analysis.

### OT-I and OT-II T cell proliferation

CD4 T cells from OT-II mice or CD8 T cells from OT-I mice were isolated from spleens using CD4 T cell or CD8 T cell isolation kits (Miltenyi Biotec), respectively. The cells were suspended in PBS/0.1% BSA solutions containing 10 μM of CFSE (Invitrogen) and incubated for 10 min. CFSE-labeled cells (1 × 10^6^) were *i.v*. transferred into CD45.1 congenic mice, and 24 hours later, the mice were injected with PBS alone, 50 μg of OVA in PBS, 25 mg/kg of laminarin, and the combination of laminarin and OVA in PBS. At 72 hours after treatment, splenocytes were harvested and OT-I or OT-II T cell proliferation was determined by analyzing the CFSE fluorescence intensity using flow cytometry.

### *In vivo* cytotoxicity assay

Mice were injected *i.v*. with a mixture of splenocytes differentially labeled with CFSE (200 nM) and loaded with 100 nM of SIINFEKL peptide, and spleen cells were labeled with 10 mM of CellTracker™ Orange CMTMR (Life technologies) and not loaded with a peptide. Then, 10 × 10^6^ cells were injected per mouse, consisting of a mixture containing each target cell population. Splenocytes were collected 24 hours after injection of the target cells. The percentage of cancer cells killed was calculated using the formula described in previous research [50].

### Intrasplenic B16-OVA injection

C57BL/6 mice were anesthetized with a ketamine mixture (10 μL of ketamine HCl, 7.6 μL of xylazine, 2.4 μL of acepromazine maleate, and 10 μL of H_2_O) injected into the peritoneal cavity. B16-OVA melanoma cells (0.5 × 10^6^/50 μL) were injected into the spleen of the mice during open laparotomy for experimentation.

### ELISPOT assay

Mouse IFN-γ ELISPOTs were performed according to the manufacturer's protocol (Biolegend). In short, spleens were harvested from treated mice and mononuclear cells were isolated by density cuts. The cells were seeded at 50 × 10^3^ cells/well in a pre-coated plate. The cells were stimulated with 2 μg/mL of OVA peptide (257–264; SIINFEKL) and OVA (323-339; ISQAVHAAHAEINEAGR) or a negative control peptide at 37°C for 24 hours. ELISPOT plates were counted automatically using a CTL ELISPOT reader (CTL Europe GmbH, Bonn, Germany), and the number of spots observed with a control peptide was subtracted from the number of spots observed with specific peptides for each mouse.

### Statistical analysis

Results were expressed as the mean ± standard error of the mean (SEM). Data sets were analyzed using a one-way ANOVA and a Tukey multiple comparison test using GraphPad Prism 4. P values smaller than 0.05 were considered to be statistically significant.

### Data availability

The data supporting the findings of this study are available from the corresponding author upon reasonable request.

## SUPPLEMENTARY FIGURE



## Abbreviations

DC: dendritic cell
cDC: conventional DC
Ag: antigen
APC: Ag presenting cell
CTL: cytotoxic T lymphocyte
Th: T helper
MHC: major histocompatibility complex
IL: interleukin
TNF: tumor necrosis factor
IFN: interferon
OVA: ovalbumin
