# Supplementary material for: Laminarin promotes anti-cancer immunity by the maturation of dendritic cells
Source: Oncotarget. 2017 Mar 14;8(24):38554–67. doi: 10.18632/oncotarget.16170 (PMC5503553; doi:10.18632/oncotarget.16170)
Supplement: Supplementary file 1 [file oncotarget-08-38554-s001.pdf]

# Laminarin promotes anti-cancer immunity by the maturation of dendritic cells

## SUPPLEMENTARY MATERIALS

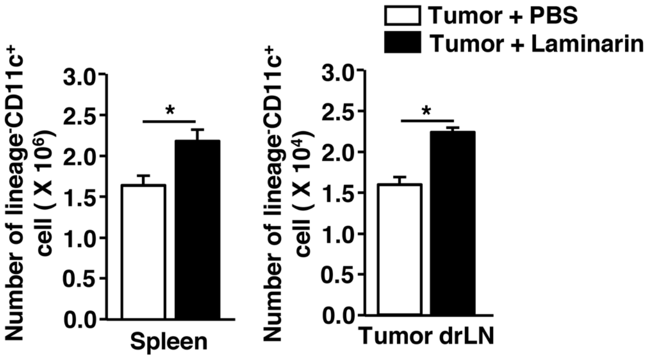


**Supplementary Figure 1: Laminarin induced increases in the DC numbers in spleen and tumor drNL in the tumor-bearing mice.** C57BL/6 mice were injected subcutaneously (*s.c.*) with 1 X $10^6$ B16 melanoma cells. On day 10 after tumor cell injection, once tumors were well established, the mice were treated with 25 mg/kg ascophyllan for 24 hours and spleen and tumor draining lymph node (drLN) were harvested. Percentages of lineage$^-$CD11c$^+$ DCs in spleen and tumor drLN were analyzed on a flow cytometry. Data are the average of analyses of 4 independent samples (2 mice per experiment, total 2 independent experiments). *$p < 0.05$.
